# Bioactivity and Metabolomic Profile of Extracts Derived from Mycelial Solid Cultures of *Hypsizygus marmoreus*

**DOI:** 10.3390/microorganisms11102552

**Published:** 2023-10-13

**Authors:** Paola Angelini, Giancarlo Angeles Flores, Gaia Cusumano, Roberto Venanzoni, Roberto Maria Pellegrino, Gokhan Zengin, Simonetta Cristina Di Simone, Luigi Menghini, Claudio Ferrante

**Affiliations:** 1 Department of Chemistry, Biology and Biotechnology, University of Perugia, 06122 Perugia, Italy; giancarlo.angelesflores@unich.it (G.A.F.); gaia.cusumano@studenti.unipg.it (G.C.); roberto.venanzoni@unipg.it (R.V.); roberto.pellegrino@unipg.it (R.M.P.); 2 Physiology and Biochemistry Research Laboratory, Department of Biology, Science Faculty, Selcuk University, 42130 Konya, Turkey; gokhanzengin@selcuk.edu.tr; 3 Botanic Garden “Giardino dei Semplici”, Department of Pharmacy, “Gabriele d’Annunzio” University, Via dei Vestini 31, 66100 Chieti, Italy; simonetta.disimone@unich.it (S.C.D.S.); claudio.ferrante@unich.it (C.F.)

**Keywords:** *Hypsizygus marmoreus*, medicinal mushroom, antimicrobial activity, antioxidant activity, metabolomics

## Abstract

The beech mushroom (*Hypsizygus marmoreus*) is a highly nutritious, edible medicinal mushroom native to East Asia. The present research investigated the impact of different substrates on the metabolite compositions of *H. marmoreus* mycelia cultivated in vitro. The substrates tested included malt extract agar, malt extract agar enriched with barley malt, and malt extract agar enriched with grape pomace. The study also assessed antimicrobial and antiradical activities of the extracts against gram-positive bacteria (*Bacillus subtilis* and *Staphylococcus aureus*), gram-negative bacteria (*Escherichia coli*, *Salmonella typhi*, and *Pseudomonas aeruginosa*), yeasts (*Candida albicans*, *C. tropicalis*, and *C. parapsilosis*), and dermatophytes (*Trichophyton mentagrophytes*, *T. tonsurans*, *T. rubrum*, *Arthroderma quadrifidum*, *A. gypseum*, *A. curreyi*, and *A. insingulare*). The results revealed that the *H. marmoreus* mycelia extracts demonstrated antibacterial and antifungal activities against the tested microorganisms. Extracts obtained from the cultivation in substrates enriched with either barley malt or grape pomace exhibited the highest antibacterial activity among all the tested bacterial strains except for *P. aeruginosa*. The same extracts showed the highest inhibitory effect against *C. albicans* and *C. parapsilosis*. Noteworthy, the extract from the mushroom cultivated in the substrate enriched with grape pomace also exhibited remarkable efficacy against *T. mentagrophytes* and *T. tonsurans.* Terpenoid and carbapenem compounds could be related to the antimicrobial properties of the extracts from mushrooms cultivated in substrates enriched with grape pomace. In comparison, the higher antiradical properties could be related to the content of indole compounds. In conclusion, growth substrate selection affects the nutritional and medicinal properties of *H. marmoreus*, making it a valuable contribution to the understanding of the cultivation of this mushroom.

## 1. Introduction

The nutritional and health-promoting properties of mushrooms have been known for thousands of years. Ancient civilizations in China, Japan, and other eastern countries have long recognized the various biological activities of mushroom extracts [1,2]. Edible mushrooms are valued for their high protein levels, dietary fiber, vitamins, minerals, phenolic compounds, and low fat [1,3]. Moreover, certain edible mushrooms contain numerous bioactive compounds that offer therapeutic benefits [4,5,6].

*Hypsizygus marmoreus* (Peck) H.E. Bigelow is well known for its nutritional and medicinal properties, among which are antitumor, antibacterial, antifungal, anti-inflammatory, antioxidant, antihypertensive, and antiallergic properties [7,8,9,10]. *H. marmoreus* belongs to the *Hypsizygus* genus (*Lyophyllaceae* family) and includes four other species: *H. elongatipes* (Peck), H.E. Bigelow, *H. ulmarius* (Bull.) Redhead, *H. tessulatus* (Bull.) Singer, and *H. ligustri* Raithelh [11].

It can be found in different countries in North America, Asia, and Europe (among which Italy) [12]. It thrives in beech stumps, withered maple, and other trees [13]. Globally, there are less than 150 known records of this species, and it is not indexed as threatened in the IUCN Red List of Endangered Species. Even in Italy, *H. marmoreus* is considered a rare species.

In addition to their well-known antioxidant activity, phenolic compounds found in mushrooms can exhibit significant antimicrobial effects against a wide range of organisms [10]. For instance, Oka et al. [14] investigated the antimicrobial activity of the volatile chemicals synthesized by *H. marmoreus* against eight phytopathogenic fungi, reporting significant inhibitory effects on Alternaria brassicicola. Shiono et al. [15] isolated and studied three polyacetylene compounds from the organic extract of *H. marmoreus*, finding that one exhibited antifungal activity against the pathogenic fungus *Raffaelea quercivora*. Wong et al. [16] evaluated different polarity extracts from *H. marmoreus*, observing their inhibition effects on *Candida albicans*. The medicinal properties of *H. marmoreus* are attributed to several bioactive molecules, including terpenoid compounds like hypsiziprenol A9, which have been shown to exert cytotoxic effects in human liver cancer HepG2 cells [17]. The dietary effects of *H. marmoreus* are associated with various active components, mainly primary metabolites [8]. As a result of its multiple uses, the production and consumption of this medicinal mushroom have been increasing worldwide. *H. marmoreus*, known as a white-rot fungus [18], possesses the ability to degrade lignocellulosic materials and can be cultivated on biological, agricultural, or agro-industrial wastes. Studies have identified various suitable substrates for mushroom cultivation, including rice straw, maize, oak wood, horse chestnut, sawdust, and cotton stalks [19]. Edible mushroom cultivation is considered a biotechnological process that helps reduce and protect the environment from excessive solid waste, as stated by Sanchez [20]. Previous studies have demonstrated that the production of cultivated mushrooms can be influenced by the fruiting body or mycelium production processes, with chemical changes affecting their pharmacological effects on health [21]. Solid culture methods for fruiting body production typically require a lengthy period, thus making it important to optimize in vitro mycelium cultures with the final goal of developing innovative health-promoting products [22]. Moreover, mycelium can be produced in a compact space within a shorter timeframe and with minimal risk of contamination [23]. The interest in the pharmaceutical potential of mushrooms has surged in the past decade, as mushrooms are regarded as small pharmaceutical farm for the synthesis of compounds with potential health effects.

In this study, our main objectives were as follows: (a) selecting two different solid media for cultivating *H. marmoreus* mycelium based on preliminary experiments, (b) investigating and comparing the chemical profiles of *H. marmoreus* mycelium grown on these two different culture media, and (c) exploring the influence of medium ingredients on the antimicrobial and antiradical activities. To achieve these goals, we employed mass spectrometry (MS)-based metabolomics, which enables quantitative analyses with high selectivity and sensitivity, along with the potential to identify metabolites [24]. Combining this approach with multivariate statistical analysis will facilitate the determination of the effects of different culture media on metabolomics and transcriptomic disparities in *H. marmoreus* mycelia.

## 2. Materials and Methods

### 2.1. Mushrooms

*H. marmoreus* (fruiting body) was collected in Perugia (via Roma) in December 2018. The voucher specimen (PeruMyc2422) was deposited in the herbarium of the Department of Chemistry, Biology, and Biotechnology (University of Perugia, Perugia, Italy). The isolation of the mycelia was carried out as previously described [3].

### 2.2. Molecular Identification

Genomic DNA was extracted from 15-day mycelia grown on malt extract agar (MEA) using the ZR Fungal/Bacterial DNA kit (Euroclone S.p.A., Milan, Italy). The amplification of the internal transcribed spacer (ITS) region was conducted with the fungal-specific forward primer, ITS1F 5′-CTTGGTCATTTAGAAGTAA-3′ in combination with the reverse primer, ITS4 5′-TCCTCCGCTTATTGATATGC-3′ [25]. The conditions for PCR amplification are included in our previous study [3].

### 2.3. Phylogeny

The chromatogram was examined and edited using Chromas 2.6.6, while MEGA X software was employed for the subsequent sequence analysis. For the phylogenetic inference, the DNA sequences of *H. marmoreus*, *Hypsizygus tessulatus* (Bull.) Singer, and *Hypsizygus ulmarius* (Bull.) Redhead were retrieved from GenBank. Additionally, Ossicaulis lignatilis (Pers.) Redhead and Ginns was chosen as an outgroup for the ITS region analysis. Within the MEGA X package, the MUSCLE algorithm was utilized for pairwise and multiple sequence alignments, and the resulting alignments were further refined manually. To support the branches in the maximum likelihood (ML) analysis, a bootstrap (BS) value of 1000 pseudoreplicates was applied.

### 2.4. Samples’ Preparation

The in vitro cultivation of *H. marmoreus* was carried out using the following solid media: (1) malt extract agar 1%, as the *Hypsizygus* control (HC); (2) malt extract agar 1% enriched with 0.5% barley malt (HC1); (3) malt extract agar 1% enriched with 0.5% grape pomace (HC2); (4) malt extract agar 1% enriched with 2% barley malt (HC3); (5) malt extract agar 1% enriched with 2% grape pomace (HC4). The details about the preparation of the samples are fully listed in our previous study [3].

### 2.5. Total Phenolic Content

The total phenolic contents of the tested extracts were evaluated via the colorimetric method using the method described for phenolics by Acquaviva et al. [26]. The Folin–Ciocalteu assay was utilized to determine the total phenolic, and the results were expressed as gallic acid equivalent (GAE). Details are reported as Supplementary Material.

### 2.6. In Vitro Antioxidant Assays

To assess the antioxidant potential of the extracts, 2,2-Azino-bis-(3-ethyl-benzthiazoline-6-sulfonic acid) (ABTS) and 2,2-diphenyl-1-picrylhydrazyl (DPPH) assays, were used to examine the antioxidants’ ability to neutralize free radicals [27]. Each of these assays was evaluated using the Trolox equivalents (TE). Details are reported as Supplementary Material.

### 2.7. Untargeted Ultra-Performance Liquid Chromatography–Mass Spectrometry (UHPLC)-Quadrupole Time of Flight (QTOF)-Based Metabolomics and Statistical Analysis

The untargeted analysis was performed using a 1260 Infinity II LC System coupled with an Agilent 6530 Q-TOF spectrometer (Agilent Technologies, Santa Clara, CA, USA). The details are reported in our previous study [3] and available as a Supplementary Materials.

### 2.8. Antimicrobial Effects

The antimicrobial properties of the extracts were investigated as previously reported [27,28,29]. Further details can be found in the supplementary, where the employed microbial species for the tests are also listed.

## 3. Results

### 3.1. Molecular Identification of Hypsizygus marmoreus (PeruMyc2422)

**Figure 1:**
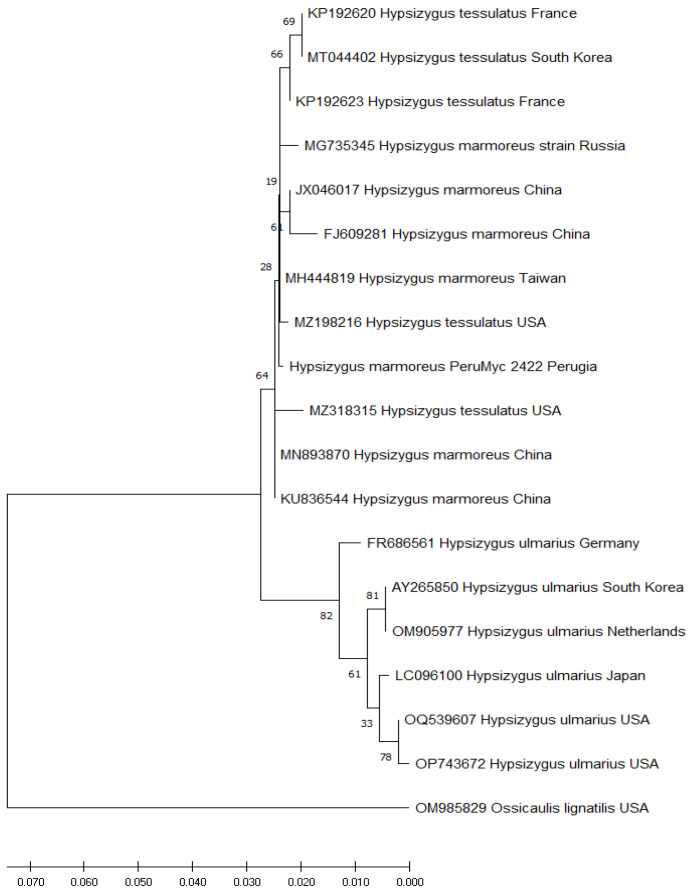
Phylogenetic tree of *Hypsizygus* species inferred using maximum likelihood (ML) based on the internal transcribed spacer (ITS) region (bootstrap support = 1000). The sequences generated in this study are represented by the Perumyc 2422 code, while the original names were maintained for sequences obtained from GenBank.

The ITS-based phylogeny provides supporting evidence for placing the newly sequenced samples from our study within the context of *H. marmoreus* and *H. tessulatus* (Figure 1). However, the sequences of *H. marmoreus* and *H. tessulatus* retrieved from GenBank in our study exhibited a high degree of genetic similarity, making it challenging to precisely classify *H. marmoreus* PeruMyc 2422 collected in Perugia. Furthermore, the alignment analysis uncovered a notable variation of four nucleotides unique to the sample we collected compared to the strains of *H. marmoreus*, *H. tessulatus*, and *H. ulmarius* obtained from GenBank. This observation highlights the importance of further investigation using additional molecular markers to gain deeper insights into the genetic differentiation within this group. Therefore, in order to enhance our understanding and achieve a more conclusive taxonomic classification, it may be necessary to conduct further investigations employing alternative molecular markers.

### 3.2. Untargeted LC–MS/MS-Based Metabolomics

Regarding the metabolomics of *H. marmoreus,* the data were analyzed with the software MS-DIAL (http://prime.psc.riken.jp/compms/msdial/main.html, accessed on 9 October 2023). The acquired information included mass, retention time, and area of the five samples analyzed (HC, H1, H2, H3, H4),

### 3.3. Statistical Data Analysis

**Figure 2:**
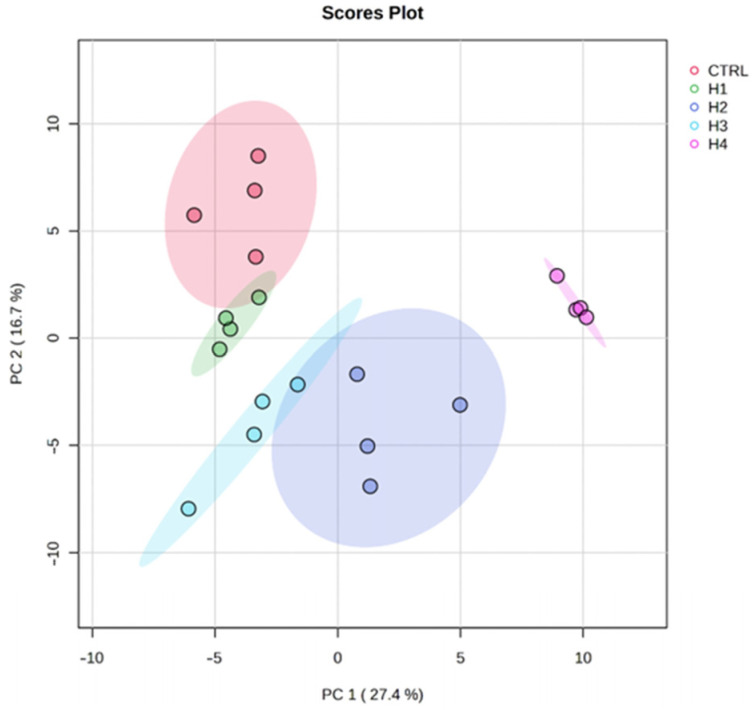
Score plot graph in which the samples were enclosed in homogenous groups (ellipsoids). Malt extract agar 1%, as the control (HC); malt extract agar 1% enriched with 0.5% barley malt (H1); malt extract agar 1% enriched with 0.5% grape pomace (HC2); malt extract agar 1% enriched with 2% barley malt (HC3); malt extract agar 1% enriched with 2% grape pomace (HC4); principal component (PC).

The data produced in this experiment were explored using the statistical technique of unsupervised principal component analysis after applying autoscaling. The score plot graph (Figure 2) shows that the first two components together explain 44.1% of the variance of the samples. The ellipsoids, which comprise the 95% confidence interval, enclose the samples in homogeneous groups. The first component separates control (HC), H1, and H3 groups from H2 and H4. The second component separates HC and H4 from everything else.

### 3.4. Cluster Analysis

**Figure 3:**
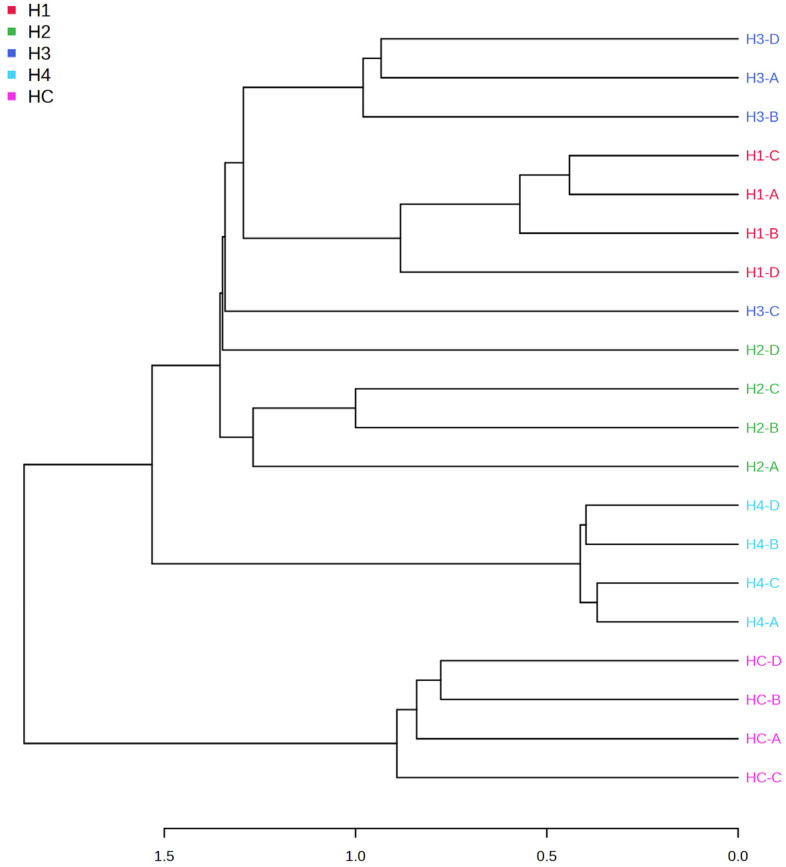
Dendrogram that shows the five *H. marmoreus* samples (HC, H1, H2, H3, and H4) divided into two main clusters. Malt extract agar 1%, as the control (HC); malt extract agar 1% enriched with 0.5% barley malt (H1); malt extract agar 1% enriched with 0.5% grape pomace (HC2); malt extract agar 1% enriched with 2% barley malt (HC3); malt extract agar 1% enriched with 2% grape pomace (HC4).

**Figure 4:**
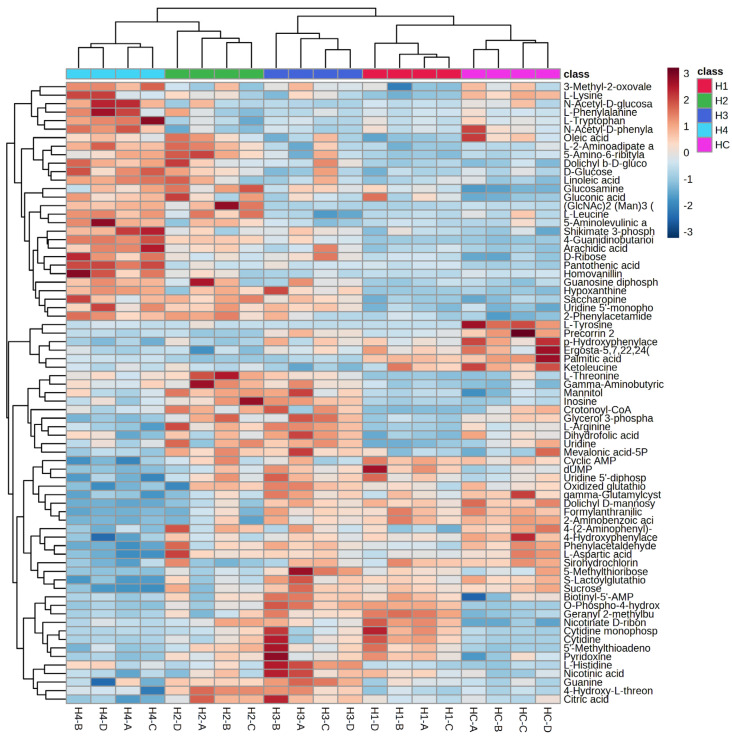
Heatmap reports the metabolomics analysis results of the two main clusters into which the samples are divided. Malt extract agar 1%, as the control (HC); malt extract agar 1% enriched with 0.5% barley malt (H1); malt extract agar 1% enriched with 0.5% grape pomace (HC2); malt extract agar 1% enriched with 2% barley malt (HC3); malt extract agar 1% enriched with 2% grape pomace (HC4).

The dendrogram (Figure 3) shows that the samples are mainly divided into two clusters: HC (fungus grown in control medium) and all the other samples grown in the samples admixed with different concentrations of barley malt (H1 and H3) or grape pomace (H2 and H4). Further nodes of the dendrogram subdivide the samples into their respective clusters. A heatmap (Figure 4) was also made considering the 70 most significant metabolites. The heatmap confirms that the samples are mainly divided into two clusters. The first node divides the HC clusters, H1 and H3, from the H2 and H4 clusters. This is consistent with the different substrates employed for the mushroom cultivation. Indeed, samples H1 and H3 were grown in a barley malt-enriched medium, while samples H2 and H4 were grown in a pomace-enriched medium.

### 3.5. Functional Analysis

**Figure 5:**
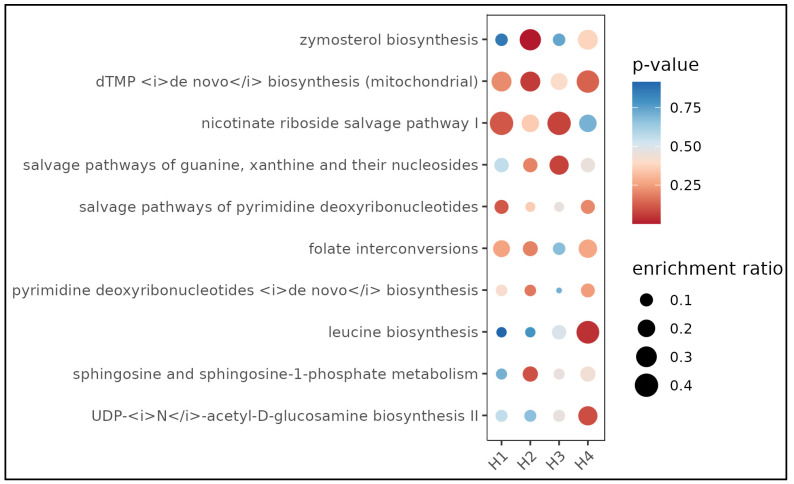
Statistically significant pathways activated with respect to control (HC) and in relationships with the employed substrate.

**Figure 6:**
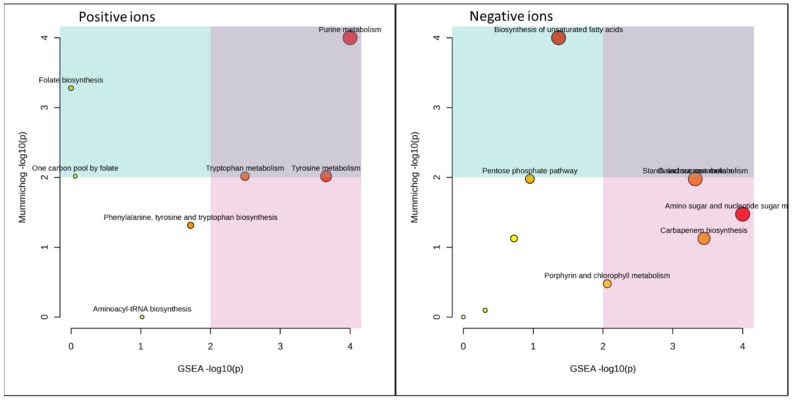
Barley malt vs. control (HC): functional analysis of untargeted metabolomics data. GSEA: gene set enrichment analysis. Mummichog: American–Indian term for by-groups.

**Figure 7:**
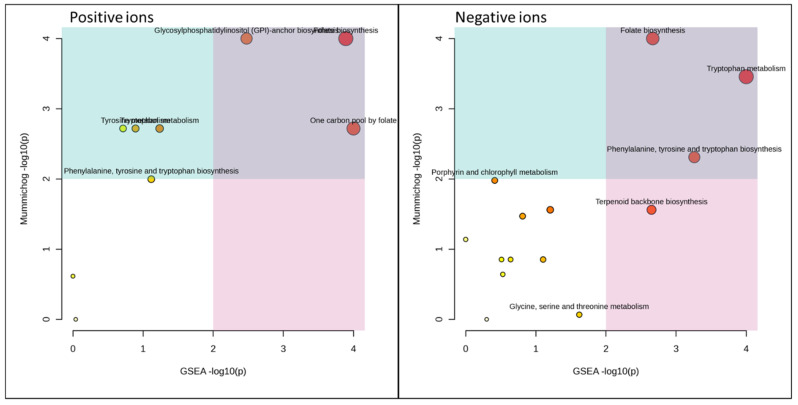
Grape pomace vs. control (HC): functional analysis of untargeted metabolomics data. GSEA: gene set enrichment analysis.

Figure 5, Figure 6 and Figure 7 and Table 1 and Table 2 summarize the MetaboAnalyst’s functional meta-analysis result. The 10 pathways activated in a statistically significant way with respect to control (HC) are reported in relationships with the employed substrate. In many cases, it is noted that the H1 and H3 pairs and H2 activate certain pathways in a similar way. For example, zymosterol biosynthesis appears activated in H2 and H4 but not in H1 and H3, thus demonstrating the substrate’s effect on mushroom metabolism [3].

### 3.6. Total Phenol Content and Antioxidant Effects

The extracts from *H. marmoreus* were also tested for the amounts of phenolic compounds, calculated as gallic acid equivalents (Table 3). However, we have to consider that the determination of total phenols through the Folin–Ciocalteu assay is a rough approximation, thus not excluding the presence of other antioxidant compounds. The results showed that substrate did not influence the total phenol content in most of the samples, with the exception of the H4 extract, which displayed a 24.28% reduction in total phenols. This is consistent, albeit partially, with its lower activity in the DPPH assay (Table 4). On the other hand, in the ABTS assay, we observed a sensitive increase in the antiradical activity, especially in the extract H4 (Table 5) derived from mushrooms cultivated in substrates with the highest concentration in grape pomace (2%). The discrepancies observed between the antiradical activity, expressed as Trolox equivalents, with DPPH and ABTS assays may be related to a lower sensitivity of DPPH to the change of growth substrate [3]. This is consistent with the higher accuracy of ABTS in measuring the antioxidant activity of extracts rich in lipophilic and highly pigmented compounds [30]. Antioxidant properties of *H. marmoreus* have been reported in the literature [31]. It is also sensitive to highlight the recent identification of indole compounds in extracts from *H. marmoreus* [32]. Considering the antioxidant properties of such compounds, we cannot exclude that the highest antioxidant effect of extract H4, as shown by ABTS assay, could be related to the presence of indole compounds [33,34]. Indeed, the H4 extract showed the highest content in indole compounds, as revealed by the metabolomics investigation (Figure 3).

### 3.7. Antimicrobial Activity

Table 6 presents the minimal inhibitory concentration (MIC) values of extracts from *H. marmoreus* mycelia against bacteria, yeasts, and dermatophytes. All extracts derived from *H. marmoreus* displayed antimicrobial activity in concentrations ranging from 1.56 to 200 μg mL^−1^. Notably, *Escherichia coli* (PeruMycA 2) exhibited the highest sensitivity to the H4 extract, with an MIC range of 1.56–3.125 μg mL^−1^ (GM, 2.48 μg mL^−1^). In contrast, *Bacillus cereus* (PeruMycA 4) and *Pseudomonas aeruginosa* (ATCC 15442) showed the least sensitivity to the mycelia extracts. Generally, gram-negative bacteria (*E. coli* PeruMyc 2 and 3, *S. typhi* 7, and *P. aeruginosa* ATCC 15442) displayed lower sensitivity to the extracts compared to gram-positive strains, similar to observations for *F. torulosa* and *Pleurotus* spp. [2,3,35]. The growth inhibition results for yeast strains revealed significant, albeit partial, antimicrobial activity of the mycelia extracts derived from the growth substrate containing grape pomace. Notably, the mycelia extracts H1 and H4 exhibited strong inhibition (MIC 12.5–100 μg mL^−1^) against *Candida albicans* (YEPGA 6379) and *C. parapsilosis* (YEPGA 6551), respectively (Table 7). Additionally, all tested extracts effectively inhibited dermatophyte growth, with *Trichophyton mantagrophytes* (CCF 4823), *T. tonsurans* (CCF 4834), and *T. rubrum* (CCF 4933) being the most susceptible fungal species to all mycelia extracts, with MIC ranging from 6.26 to 200 μg mL^−1^. MIC values below 100 μg mL^−1^ were considered indicative of high antimicrobial activity (Table 8). Regarding the mechanisms underlying the observed antimicrobial effects, according to the metabolomics analysis, we hypothesize that different secondary metabolites, including terpenoids and carbapenems, could be responsible for the antibacterial and antifungal effects of the extracts. In particular, the contemporary presence of terpenoids and carbapenems in the extracts prepared from mushrooms cultivated in the medium enriched with grape pomace further corroborates the overall higher efficacy of the H4 as an antimicrobial. However, it cannot exclude that other mushroom metabolites, including sterols and polyisoprenepolyols could be involved in the antimicrobial properties displayed by *H. marmoreus* [36].

## 4. Conclusions

In the present study, the influence of different substrates was investigated on the metabolomics profile and biological properties of extracts from the medicinal mushroom *H. marmoreus*. The supplementation of the agar substrate with grape pomace determined a significant increase in the antioxidant and antimicrobial properties of the mushroom. Although a wide plethora of studies indicated the phenolic compounds as key actors in the antimicrobial and antioxidant effects from polar extracts of both medicinal plants and mushrooms, in the present study antiradical and antimicrobial effects were not related to the content of phenolics. On the other hand, the metabolomics approach showed significant effects of the growth substrates on the mushroom pathways, suggesting that the enrichment of the growth medium with grape pomace could be an innovative strategy to implement the medicinal properties of *H. marmoreus*, whose content in terpenoids, carbapenems, and indoles was significantly increased compared with the mushrooms cultivated in the standard medium. Future studies are needed to confirm the observed antioxidant and antimicrobial effects in in vivo pharmacological models. However, as a concluding remark, these results, besides showing an innovative approach to modulate the metabolic pathways’ activation and consequent biopharmacological properties of the mushroom, also represent a paradigm of the possibility of integrating different productive chains, medicinal mushrooms (i.e., *H. marmoreus*), and medicinal plants (i.e., *Vitis vinifera*), also in a context of sustainability through the use of by-products from a chain to feed the other one.

## Figures and Tables

**Table 1:** Statistically significantly pathways active in the barley malt samples vs. control (HC).

Barley Malt vs. Control	Total Size	Hits	Sig_Hits	Mummichog *p*-Values	GSEA *p*-Values	Combined *p*-Values
Purine metabolism	62	12	4	0.06893	0.01538	0.00832
Tyrosine metabolism	15	2	1	0.1915	0.02128	0.0265
Amino sugar and nucleotide sugar metabolism	22	6	1	0.1549	0.03509	0.03378
Biosynthesis of unsaturated fatty acids	23	3	2	0.0228	0.2419	0.0342
Galactose metabolism	17	7	2	0.1056	0.05769	0.03718
Starch and sucrose metabolism	12	5	3	0.1056	0.05769	0.03718
Carbapenem biosynthesis	3	2	1	0.2018	0.05263	0.0589
Tryptophan metabolism	30	2	1	0.1915	0.06383	0.06607
Pentose phosphate pathway	18	2	1	0.1056	0.3269	0.1508
Phenylalanine, tyrosine, and tryptophan biosynthesis	21	3	1	0.2752	0.1333	0.1579
Porphyrin and chlorophyll metabolism	20	5	1	0.3298	0.1452	0.1934
Folate biosynthesis	23	1	1	0.1	0.6735	0.249
Pentose and glucuronate interconversions	12	4	2	0.2018	0.386	0.2767
One carbon pool by folate	8	2	1	0.1915	0.6364	0.3784
Aminoacyl-tRNA biosynthesis	22	7	1	0.5414	0.2571	0.4137
Glycine, serine and threonine metabolism	28	9	1	0.4403	0.5224	0.568
Arginine and proline metabolism	25	9	2	0.4735	0.6567	0.6742

Hits: number of identified metabolites that are part of a specific pathway; Sig_Hits: number of identified metabolites that are part of a specific pathway, whose concentration has varied in a statistically significant manner; mummichog: American–Indian term for by-groups; GSEA: gene set enrichment analysis.

**Table 2:** Statistically significantly pathways active in the grape pomace samples vs. control (HC).

Grape Pomace vs. Control	Total Size	Hits	Sig_Hits	Mummichog *p*-Values	GSEA *p*-Values	Combined *p*-Values
Amino sugar and nucleotide sugar metabolism	22	6	1	0.2487	0.9375	0.5726
Aminoacyl-tRNA biosynthesis	22	7	1	0.4746	0.8529	0.7708
Arginine and proline metabolism	25	9	2	0.6641	0.6818	0.8116
Carbapenem biosynthesis	3	2	1	0.3183	0.2889	0.3114
Folate biosynthesis	23	2	1	0.02103	0.05455	0.00891
Galactose metabolism	17	7	2	0.1728	0.2593	0.184
Glycine, serine and threonine metabolism	28	9	1	0.6272	0.1667	0.3407
Glycosylphosphatidylinositol (GPI)-anchor biosynthesis	11	1	1	0.08333	0.07547	0.03817
N-Glycan biosynthesis	28	3	2	0.161	0.4237	0.2514
One carbon pool by folate	8	2	1	0.161	0.01695	0.01884
Pantothenate and CoA biosynthesis	18	4	1	0.3183	0.4737	0.436
Pentose and glucuronate interconversions	12	4	1	0.3183	0.5439	0.4767
Phenylalanine, tyrosine, and tryptophan biosynthesis	21	4	1	0.09046	0.02899	0.01821
Porphyrin and chlorophyll metabolism	20	5	2	0.1204	0.6029	0.263
Purine metabolism	62	12	1	0.6508	0.8205	0.869
Starch and sucrose metabolism	12	5	3	0.1728	0.2593	0.184
Terpenoid backbone biosynthesis	13	2	1	0.1728	0.05556	0.05421
Tryptophan metabolism	30	7	2	0.0336	0.01316	0.00386
Tyrosine metabolism	15	2	1	0.161	0.3559	0.2212
Valine, leucine and isoleucine biosynthesis	20	7	2	0.1868	0.3947	0.2659
Valine, leucine and isoleucine degradation	16	5	1	0.382	0.5333	0.5278

Hits: number of identified metabolites that are part of a specific pathway; Sig_Hits: number of identified metabolites that are part of a specific pathway, whose concentration has varied in a statistically significant manner; mummichog: American–Indian term for by-groups; GSEA: gene set enrichment analysis.

**Table 3:** Total phenol content.

Sample	mg GAE (Gallic Acid Equivalents)/g_dm_	SD
H1	3.063	0.073
H2	3.418	0.400
H3	3.250	1.634
H4	2.720	0.070
HC	3.545	0.924

Malt extract agar 1%, as the control (HC); malt extract agar 1% enriched with 0.5% barley malt (H1); malt extract agar 1% enriched with 0.5% grape pomace (HC2); malt extract agar 1% enriched with 2% barley malt (HC3); malt extract agar 1% enriched with 2% grape pomace (HC4).

**Table 4:** The 2,2-diphenyl-1-picrylhydrazyl (DPPH) ASSAY.

Sample	Decolorization (%) (2 mg/mL)	SD	mg TE (Trolox Equivalents/g_dm_)	SD
H1	21.25	1.64	14.323	1.339
H2	18.92	1.06	12.428	0.862
H3	18.25	1.957	11.878	1.594
H4	18.19	3.356	11.831	2.734
HC	21.11	1.968	14.206	1.603

Malt extract agar 1%, as the control (HC); malt extract agar 1% enriched with 0.5% barley malt (H1); malt extract agar 1% enriched with 0.5% grape pomace (HC2); malt extract agar 1% enriched with 2% barley malt (HC3); malt extract agar 1% enriched with 2% grape pomace (HC4).

**Table 5:** The 2,2-Azino-bis-(3-ethyl-benzthiazoline-6-sulfonic acid) (ABTS) assay.

Decolorization (%) (2 mg/mL)	SD	mg TE (Trolox Equivalents/g_dm_)	SD
7.81	0.72	0.587	0.254
6.92	0.74	0.295	0.261
9.25	0.089	1.118	0.021
9.90	0.095	1.331	0.034
7.75	0.333	0.566	0.118

**Table 6:** Antibacterial effects of *H. marmoreus* mycelia extracts expressed as minimal inhibitory concentrations (MIC).

	MIC (µg mL^−1^)							
Bacteria	*Escherichia coli*	*Escherichia coli*	*Escherichia coli*	*Bacillus cereus*	*Pseudomonas aeruginosa*	*Bacillus subtilis*	*Salmonella typhi*	*Staphylococcus aureus*
	(ATCC 10536)	(PeruMycA 2)	(PeruMycA 3)	(PeruMycA 4)	(ATCC 15442)	(PeruMycA 6)	(PeruMycA 7)	(ATCC 6538)
Extracts								
HC	39.68 (25–50)	9.92 (6.25–12.5)	62.99 (50–100)	>200	>200	79.37 (50–100)	62.99 (50–100)	125.99 (100–200)
H1	8.29 (6.75–12.5)	3.93 (3.12–6.25)	39.68 (25–50)	>200	>200	31.49 (25–50)	31.49 (25–50)	79.37 (50–100)
H2	15.75 (12.5–25)	3.93 (3.12–6.25)	31.49 (25–50)	>200	>200	62.99 (50–100)	62.99 (50–100)	125.99 (100–200)
H3	15.75 (12.5–25)	19.84 (12.5–25)	9.92 (6.25–12.5)	>200	>200	62.99 (50–100)	31.49 (25–50)	62.99 (50–100)
H4	9.92 (6.25–12.5)	3.93 (3.12–6.25)	2.48 (1.56–3.125)	>200	>200	79.37 (50–100)	31.49 (25–50)	62.99 (50–100)
Ciprofloxacin (µg mL^−1^)	31.49 (25–50)	9.92 (6.25–12.5)	79.37 (50–100)	125.99 (100–200)	125.99 (100–200)	125.99 (100–200)	79.37 (50–100)	200–>200

Minimal inhibitory concentration (MIC) values are the geometric means of three replicates (n = 3), and the range concentrations are shown within brackets. Malt extract agar 1%, as the control (HC); malt extract agar 1% enriched with 0.5% barley malt (H1); malt extract agar 1% enriched with 0.5% grape pomace (HC2); malt extract agar 1% enriched with 2% barley malt (HC3); malt extract agar 1% enriched with 2% grape pomace (HC4).

**Table 7:** Minimal inhibitory concentrations (MIC) of *H. marmoreus* extracts against yeast isolates.

	MIC (μg mL^−1^)			
Yeast Strain	*Candida tropicalis*	*Candida albicans*	*Candida parapsilosis*	*Candida albicans*
	(YEPGA 6184)	(YEPGA 6379)	(YEPGA 6551)	(YEPGA 6183)
Extracts				
HC	79.37 (50–100)	62.99 (50–100)	158.74 (100–200)	79.37 (50–100)
H1	39.68 (25–50)	19.84 (12.5–25)	62.99 (50–100)	125.99 (100–200)
H2	39.68 (25–50)	79.37 (50–100)	39.68 (25–50)	79.37 (50–100)
H3	39.68 (25–50)	39.68 (25–50)	62.99 (50–100)	39.68 (25–50)
H4	79.37 (50–100)	79.37 (50–100)	19.84 (12.5–25)	62.99 (50–100)
Fluconazole (μg mL^−1^)	2	1	4	2

MIC values are reported as geometric means of three independent replicates (n = 3). MIC range concentrations are reported within brackets. Malt extract agar 1%, as the control (HC); malt extract agar 1% enriched with 0.5% barley malt (H1); malt extract agar 1% enriched with 0.5% grape pomace (HC2); malt extract agar 1% enriched with 2% barley malt (HC3); malt extract agar 1% enriched with 2% grape pomace (HC4).

**Table 8:** Minimal inhibitory concentrations (MICs) of *H. marmoreus* mycelia (HC) extracts against dermatophyte isolates.

	MIC (µg mL^−1^)							
Dermatophyte	*Trichophyton mentagrophytes*	*Trichophyton tonsurans*	*Trichophyton rubrum*	*Arthroderma quadrifidum*	*Trichophyton* *mentagrophytes*	*Arthroderma* *gypseum*	*Arthroderma curreyi*	*Arthroderma* *insingulare*
	(CCF 4823)	(CCF 4834)	(CCF 4933)	(CCF 5792)	(CCF 5930)	(CCF 6261)	(CCF 5207)	(CCF 5417)
Extracts								
HC	158.74 (100–200)	125.99 (100–200)	125.99 (100–200)	79.37 (50–100)	>200	>200	158.74 (100–200)	79.37 (50–100)
H1	39.68 (25–50)	31.49 (25–50)	62.99 (50–100)	79.37 (50–100)	125.99 (100–200)	125.99 (100–200)	125.99 (100–200)	62.99 (50–100)
H2	62.99 (50–100)	39.68 (25–50)	31.49 (25–50)	62.99 (50–100)	125.99 (100–200)	100–>200	100–>200	125.99 (100–200)
H3	62.99 (50–100)	31.49 (25–50)	62.99 (50–100)	79.37 (50–100)	158.74 (100–200)	158.74 (100–200)	100–>200	62.99 (50–100)
H4	19.84 (12.5–25)	9.92 (6.25–12.5)	19.84 (12.5–25)	>200	>200	62.99 (50–100)	125.99 (100–200)	79.37 (50–100)
Griseofulvin (µg mL^−1^)	2.52 (2–4)	0.198 (0.125–0.25)	1.26 (1–2)	>8	3.174 (2–4)	1.587 (1–2)	>8	>8

MIC values are reported as geometric means of three independent replicates (n = 3). MIC range concentrations are reported within brackets. Malt extract agar 1%, as the control (HC); malt extract agar 1% enriched with 0.5% barley malt (H1); malt extract agar 1% enriched with 0.5% grape pomace (HC2); malt extract agar 1% enriched with 2% barley malt (HC3); malt extract agar 1% enriched with 2% grape pomace (HC4).

## Data Availability

Original data are available from the corresponding author.

## Supplementary Materials

The following supporting information can be downloaded at https://www.mdpi.com/article/10.3390/microorganisms11102552/s1, Total Phenolic Content; 2,2-Azino-bis-(3-ethyl-benzthiazoline-6-sulfonic acid) (ABTS) and 2,2-diphenyl-1-picrylhydrazyl (DPPH) assays. Untargeted ultra-performance liquid chromatography mass spectrometry (UHPLC)-quadrupole time of flight (QTOF)-based metabolomics and statistical analysis.
