# Supplementary material for: Bioactivity and Metabolomic Profile of Extracts Derived from Mycelial Solid Cultures of Hypsizygus marmoreus
Source: Microorganisms. 2023 Oct 13;11(10):2552. doi: 10.3390/microorganisms11102552 (PMC10609027; doi:10.3390/microorganisms11102552)
Supplement: Supplementary file 1 [file microorganisms-11-02552-s001.zip › microorganisms-2612456-supplementary.pdf]

Total Phenolic Content

The total phenolic content was determined by employing the methods in the literaturewith some modifications. A sample solution (0.25 mL) was mixed with a diluted Folin–Ciocalteu reagent (1 mL, 1:9, v/v) and shaken vigorously. After 3 min, the Na_2CO_3 solution (0.75 mL, 1%)was added, and the sample absorbance was read at 760 nm after a 2 h incubation at room temperature. The total phenolic content was expressed as milligrams of gallic acid equivalents (mg GAE/g extract).

DPPH and ABTS assays

For the DPPH (1,1-diphenyl-2-picrylhydrazyl) radical scavenging assay, a sample solution was added to 4 mL of a 0.004% methanol solution of DPPH. The sample absorbance was read at 517 nm after a 30 min incubation at room temperature in the dark. DPPH radical scavenging activity was expressed as milligrams of Trolox equivalents (mg TE/g extract).

For the ABTS (2,2′-azino-bis(3-ethylbenzothiazoline) 6-sulfonic acid) radical scavenging assay: Briefly, ABTS+ was produced directly by reacting a 7 mM ABTS solution with 2.45 mM potassium persulfate and allowing the mixture to stand for 12–16 in the dark at room temperature. Prior to beginning the assay, the ABTS solution was diluted with methanol to an absorbance of 0.700 ± 0.02 at 734 nm. The sample solution was added to the ABTS solution (2 mL) and mixed. The sample absorbance was read at 734 nm after a 30 min incubation at room temperature. The ABTS radical scavenging activity was expressed as milligrams of Trolox equivalents (mg TE/g extract).

Untargeted Ultra-Performance Liquid Chromatography–Mass Spectrometry (UHPLC)-Quadrupole Time Of Flight (QTOF)-Based Metabolomics and Statistical Analysis

Untargeted analysis was performed using a 1260 Infinity II LC System coupled with an Agilent 6530 Q-TOF spectrometer (Agilent Technologies, Santa Clara, CA, USA). The LC comprises a quaternary pump, a thermostated column compartment, and an autosampler. Separation was carried out on an Agilent InfinityLab Poroshell 120 HILIC-Z, 2.1 ⇥ 150 mm, 2.7 μm at 25 °C, and 0.25 mL/min flow. The mobile phase consisted of a mixture of water (A) and water/acetonitrile 15:85 (B), both containing a concentration of 10 mM ammonium acetate. The gradient was: time 0–3 min isocratic at A 2%, B 98%; time from 3 to 11 min: linear-gradient to A 30%, B 70; time 11–12 min linear gradient to A 60%, B 40%; time from 12 to 16 min: linear-gradient to A 95%, B 5%; time 16–18 min isocratic at A 95%, B 5%; time 18 min: stop run. Spectrometric data were acquired in the 40–1700 m/z range, both in negative and positive polarity. The Agilent Jet Stream source operated as follows: Gas Temp (N2) 200 C, Drying Gas 10 L/min, Nebulizer 50 psi, and Sheath Gas temp: 300 °C at 12 L/min. Raw data were processed using MS-DIAL software (4.48) to perform peak-picking, alignment, and peak integration. The mass spectrometer signal threshold was set at 1000 counts. Finally, a data matrix was obtained reporting the accurate mass and area of each peak revealed in each sample analyzed. The putative annotation of metabolites and the prediction of metabolic pathways was performed using the mummichog algorithm, implemented in the 'MS Peaks to Pathways' module of Metaboanalyst 5.0. It considers any possible adducts and different ionic polarities and classifies the peaks annotated on the basis of the t-test. In this case, the list of putative compounds was mapped onto the KEGG library of *Saccaromices cerevisiae*. Analysis of variance (ANOVA) and functional meta-analysis were also performed with MetaboAnalyst. For statistical analysis, samples were normalized via the median,

followed by Pareto scaling.

Antimicrobial Effects

The in vitro antimicrobial activity of extracts was evaluated against the following Gram-negative and Gram-positive bacterial strains: *Escherichia coli* (American Type Culture Collection, ATCC 10536), *E. coli* (PeruMycA 2), *E. coli* (PeruMycA 3), *Bacillus cereus* (PeruMycA 4), *B. subtilis* (PeruMyc 6), *Salmonella typhy* (PeruMyc 7), *Pseudomonas aeruginosa* (ATCC 15442), and *Staphylococcus aureus* (ATCC 6538). Additionally, the same extracts were tested for their antifungal activity against different yeasts, dermatophyte, and fungal pool species: *Candida albicans* (Yeast Collection of Perugia, YEPGA 6183), *C. tropicalis* (YEPGA 6184), *C. albicans* (YEPGA 6379), *C. parapsilopsis* (YEPGA 6551), *Arthroderma crocatum* (Culture Collection of Fungi, CCF 5300), *A. curreyi* (CCF 5207), *A. gypseum* (CCF 6261), *A. quadrifidum* (CCF 5792), *A. insingulare* (CCF 5417), *A. quadrifidum* (CCF 5792), *Trichophyton mentagrophytes* (CCF 4823), *T. mentagrophytes* (CCF 5930), *T. rubrum* (CCF 4933), and *T. tonsurans* (CCF 4834).
